# Ethnomedicinal, Phytochemical and Ethnopharmacological Aspects of Four Medicinal Plants of Malvaceae Used in Indian Traditional Medicines: A Review

**DOI:** 10.3390/medicines4040075

**Published:** 2017-10-18

**Authors:** Jasmeet Kaur Abat, Sanjay Kumar, Aparajita Mohanty

**Affiliations:** 1 Department of Botany, Gargi College, Sirifort Road, New Delhi 110049, India; jasmeet29@yahoo.com; 2 Department of Microbiology, Maharshi Dayanand University, Rohtak, Haryana 124001, India; sanjay.micro@mdurohtak.ac.in

**Keywords:** *Abutilon indicum*, *Hibiscus sabdariffa*, *Sida acuta*, *Sida rhombifolia*, ethnopharmacological, Malvaceae, phytoconstituents

## Abstract

The ethnomedicinal values of plants form the basis of the herbal drug industry. India has contributed its knowledge of traditional system medicines (Ayurveda and Siddha) to develop herbal medicines with negligible side effects. The World Health Organization has also recognized the benefits of drugs developed from natural products. *Abutilon indicum*, *Hibiscus sabdariffa*, *Sida acuta* and *Sida rhombifolia* are ethnomedicinal plants of Malvaceae, commonly used in Indian traditional system of medicines. Traditionally these plants were used in the form of extracts/powder/paste by tribal populations of India for treating common ailments like cough and cold, fever, stomach, kidney and liver disorders, pains, inflammations, wounds, etc. The present review is an overview of phytochemistry and ethnopharmacological studies that support many of the traditional ethnomedicinal uses of these plants. Many phytoconstituents have been isolated from the four ethnomedicinal plants and some of them have shown pharmacological activities that have been demonstrated by in vivo and/or in vitro experiments. Ethnomedicinal uses, supported by scientific evidences is essential for ensuring safe and effective utilization of herbal medicines.

## 1. Introduction

Herbal drugs from ethnomedicinal plants have gained considerable importance in the recent past not only in India but also around the world [1]. Traditional medicinal knowledge in India has passed from one generation to the next, within specific geographical locations or tribal groups [2]. This traditional knowledge finds its root in Indian traditional systems of medicine i.e., Ayurveda and Siddha which is now gaining popularity in western world too. Ethnomedicines/herbal medicines are much in demand as they are affordable and have much less side effects [3]. Recently WHO has also recognized the importance of traditional medicine in the healthcare sector [4,5]. In Ayurveda and Siddha systems, formulations from appropriate parts of plants are made and used for treatment of various ailments. For almost past three decades, many ethnomedicinal plants mentioned in Ayurveda and Siddha systems of medicines are being scientifically evaluated [6]. Scientific evaluation of ethnomedicinal plants, provides evidence-based alternative medicines which form the basis of herbal drug industry and discovery of drug targets in the pharmaceutical industry [7]. It maybe emphasized here that usage of ethnomedicinal plants for traditional medical treatment or for use in manufacture of Ayurvedic medicines/other herbal drugs, when supported by scientific evidences can ensure safe and more effective utilization of natural product drugs universally.

Malvaceae family encompasses approximately 244 genera with 4225 species of herbs, shrubs and trees [8]. Around 22 genera of the family are reported from India, many of which have ethnomedicinal value e.g., *Abutilon indicum*, *Gossypium herbaceum*, *Hibiscus mutabilis*, *Hibiscus sabdariffa*, *Hibiscus rosa-sinensis*, *Sida acuta*, *Sidacordifolia*, *Sida rhombifolia* and several others [9]. Keeping this in viewthe ethnomedicinal, phytochemical and ethnopharmacological aspects of four Malvaceae members (*Abutilonindicum*, *Hibiscus sabdariffa*, *Sida acuta*, *Sida rhombifolia*), used as crude herbal drugs by ethnic tribes in India and as ingredients of Ayurvedic medicines, have been reviewed. Further, justification of traditional ethnomedicinal uses of these plants by scientific investigations including their phytochemistry and ethnopharmacological activities have been assessed. The choice of the four ethnomedicinal plants is based on the following criteria: (i) all belong to family Malvaceae; (ii) all are used in Ayurveda and Siddha system of Medicines; (iii) all are commonly used by ethnic tribes in most parts of India; and (iv) extracts of these plants are used to treat similar ailments (e.g., kidney disorders, arthritis, pains, diabetes etc.).

## 2. Ethnomedicinal Uses

### 2.1. Abutilon Indicum

*A. indicum* is referred to as ‘Atibala’ in Sanskrit language and its medicinal potential and therapeutic applications have been described in traditional Indian systems of medicines. The plant as a whole or its different parts such as leaves, flower, seed, roots, and bark have been used for treating inflammations, ulcer, diarrhea, pains, stomach ailments, diabetes and wounds [10,11,12]. Traditional practitioners used the plant to treat diseases like gout, tuberculosis, ulcer, jaundice, leprosy, gonorrhea, bronchitis, lumbago malarial fever, piles and other bleeding disorders [13,14,15,16]. Its roots and seeds are used in the form of decoction to cure fever and cough. Root of the plant have been used as nervine tonic to cure paralysis and also effective in strangury [10]. The powdered form of dried leaves of this plant mixed with wheat flour is used for treating uterus displacement among some tribes in Orissa in India [17]. Decoction of leaf and roots are used to cure dental problems [15]. There are reports of topical application of leaf paste on the spot of scorpion bite to relieve pain [18]. Flowers of this plant are used by tribal population in Southern India to increasethe volume of semen in humanbeings [19].

### 2.2. Hibiscus Sabdariffa

*H. sabdariffa* is known by the name of ‘Ambasthika’, in ancient Indian medical text. This plant, also known as roselle or sorrel (‘Gongura’ in Hindi) is used both as food and traditional medicine. Leaves, calyces, petals or whole flowers are traditionally used for their therapeutic potentials [20]. *H. sabdariffa* has been used in folk medicine as a diuretic, mild laxativeand for the treatment of kidney, cardiac and liver diseases. Different parts of the plant are also used for ailments like hypertension, pyrexia and skin inflammations [21]. In southern India, leaves, petals, fleshy calyces are used for making pickles and sour recipes which have medicinal benefits. Fruits and calyces are traditionally used by Zeliang tribe of Nagaland (north-eastern India), to treat stomach disorders, as blood purifying agent and as hair tonic [22]. In Jharkhand (eastern India), the sorrel drink is recommended for curing cough, cold and malaria [23]. Calyx decoction is used to treat poisoning by the tribes of Nandurbar district of Maharashtra (western India) [24] and as a general antidote for food and chemical poisoning by the ‘Karbis’ tribe of Assam (eastern India).

Infusion of the fleshy calyx is used as a refreshing beverage and leaves are used as diuretic by people in Salem District of Tamilnadu [25]. Powdered seed is used by Bhoxa community of Dehradun district, Uttarakhand (northern India), to treat dysentery and diarrhea [26]. In Manipur (northeastern India), tribals use a decoction of leaves to treat urinary troubles, especially kidney stones [27]. Tribes of BhiwapurTahsil of Nagpur (western India) commonly call *H. sabdariffa* as ‘ambadibhaji’ and the juice extract of the flowers with sugar and black pepper is given to treat acidity. Seed paste is externally applied to pains and bruises and leaves are used as purgative [28]. Roselleis used for treating dyspepsia and piles by tribes of Andaman and Nicobar Islands, India [29]. Also, dried calyces are used to cure menstrual disorders and other gynecological disorders [30].

Besides India, Roselle is widely utilized in other parts of the world. In African folk medicine, Roselle leaves are used for their antimicrobial, emollient, antipyretic, diuretic, anti-helminthic and sedative properties.In Nigeria, Sudan, Iran and other countries the plant is used to make a beverage/tea which is used to control blood pressure, to treat cardiac conditions, and as a diuretic [31]. Traditionally, beverages made from *H. sabdariffa* are used as cooling herb, providing relief during hot weather by increasing the flow of blood to the skin surface and dilating the pores to cool the skin [32]. The plant is used widely in Egypt for the treatment of cardiac and nerve diseases. The leaves in combination with ginger are used to prevent high blood pressure and in the treatment of hypertension [33]. Lotion made from leaf pulp is used to treat sores and external wounds [34].

### 2.3. Sida Acuta

The ethnomedicinal usage of *S. acuta* (Sanskrit name: Balapatta) has been reported from among the ethnic tribes from many parts of India. The tribal population from north eastern and southern parts of India, have been extensively using different parts of the plant for treatment of dandruff, rheumatism, liver problems, kidney stones, nervous disorders [24,25,26], testicular swelling and elephantitis [35]. Juice of fresh leaves are used as anti-helminthic, anti-vomitting and gastric disorders [36,37,38]. Paste of roots in lemon juice is applied on boils and abscises [39]. Decoction of roots is used to treat rheumatism and breathing disorders. Hot water extract of whole plant is used as diuretic [40] and for abortion of fetus [41]. Plant is also used by tribes of Tamil Nadu (Southern India) for treating bronchitis dysentery, diarrhoea and skin diseases [42]. Besides India, other Asian (Sri Lanka, Taiwan); Central and South American (Mexico, Venezuela, Colombia, Cuba, Nicaragua, Guatemala) and African countries (Nigeria, Togo, Ivory Coast, Kenya) also use this ethnomedicinal plant for treating dysentery, hemorrhoids, malaria, venereal diseases, ulcers, renal inflammations, fever and asthma [43].

### 2.4. Sida Rhombifolia

*S. rhombifolia* is known as ‘Mahabala’ in ancient text and is an ingredient of many Ayurvedic medicines used for treating inflammations, to build immunity, for well being and vitality [44]. The tribal population of many parts of India use whole plant or plant parts for treatment of piles, gout, rheumatism, kidney disorders and gonorrhoea [45]. Due to its known ethnomedicinal uses, the plant forms an important component of Ayurvedic medicines marketed as ‘Baladikwath’, ‘Baladyaghir’, ‘Baladyarishta’, ‘Sudershan Churna’ and ‘Kukuvadi churna’. These ayurvedic medicines are used to cure pain and swelling caused by rheumatism, muscular weakness, urinary tract woumds and also to treat tuberculosis, heart diseases and neurological disorders [46]. Roots of the plant are used for treating snake bites [47]. Decoction of roots is taken for rheumatic pains [48] to treat tuberculosis and also malaria [49]. Root paste is applied for healing boils [50]. Leaf of the plant in various forms is used for fever, heart disease, piles and rheumatism [44]. Fruits are used for curing headache [51].

In parts of Africa, hot aqueous extract of aerial parts of the plant is used for snake bites and abortion [52,53]. Also leaf and root extracts are used for asthma, pneumonia and bronchitis [54,55] infusion of roots is taken for treating dysentery, diarrhoea and indigestion in Australia, Cameroon and Papua New Guinea [52,55,56,57]. In Europe, roots are used for treating tuberculosis [58]. Whole plants are used for treating gout (Indonesia), irregular menses (Malaysia), fever, bodyache (Thailand), skin problems, liver problems, diarrhoea (Mexico), kidney inflammation (Bolivia), dandruff and wounds (Panama) and gonorrhoea in Guatemala [59,60,61,62,63,64,65]. In Argentina, leaves of the plants are used to treat menstrual pain [66]. Macerated leaves are orally taken for sedation, to treat hypertension and venereal diseases [55]. In Senegal and Madagascar, flowers are rubbed on wasp stings to bring relief [55].

## 3. Major Phytoconstituents

Phytoconstituents are naturally occurring chemical compounds, responsible for colour, odour and therapeutic potential of plants. Plants synthesize these compounds as weapons for defense against biotic and abiotic stresses. Most of the phytoconstituents have antioxidant properties and protect cells against oxidative stress. Phytoconstituents also have commercial applications such as drugs, enzymes, preservatives, flavors, fragrances, cosmetics and fuels. Phytochemical screening is an important tool in identifying chemical compounds of medicinal and industrial value. For screening and isolation of phytochemicals, plant parts (root, stem, leaf, etc.) and types of extraction procedure used, play crucial roles [67].

**Figure 1:**
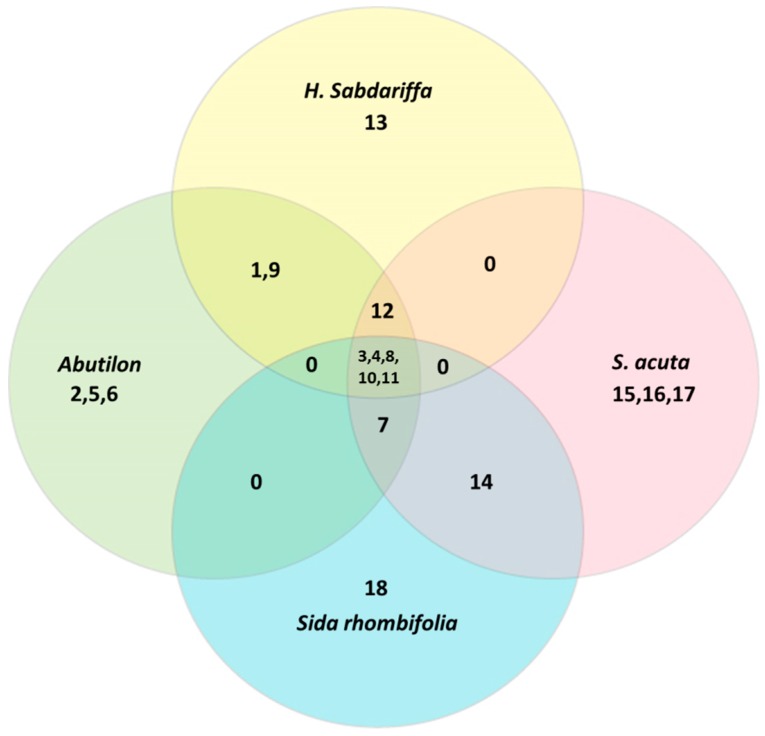
Common and unique classes of major phytoconstituents reported in *Abutilon indicum*, *Hibiscus sabdariffa*, *Sida acuta.* and *Sida rhombifolia*. Numbers 1–18 represent the class of phytochemicals, (1—Acid, 2—Alcohol, 3—Aliphatics, 4—Alkaloids, 5—Alkane hydrocarbon, 6—Aromatic ketone, 7—Coumarins, 8—Flavonoids, 9—Peptide, 10—Phenolics, 11—Steroids, 12—Tocopherols, 13—Polysaccharides, 14—Ecdysteroids, 15—Lignans, 16—Phalate, 17—Terpenoids, 18—Phaeophytins). ‘0’ represents no phytochemical class is exclusively common between any two or three plants.

There are thousands of different phytochemicals, and based on the chemical structures these are classified into various categories like alkaloids, carotenoids, phenolics, flavonoids, coumarins, steroids, tannins and others. Many phytoconstituents isolated from Malvaceae members belonging to categories such as flavonoids, phenolics, acids, and polysaccharides exhibit therapeutic activities. Table 1 lists the phytochemicals that have been reported in *A. indicum*, *H. sabdariffa*, *S. acuta* and *S. rhombifolia*. The classes of phytochemicals (e.g., alkaloids, flavonoids, phenolics) are common among the four ethnomedicinal plants and are responsible for most of the ethnopharmacological activities. Figure 1 shows the different shared and unshared classes of phytoconstituents reported among the four plants species.

### 3.1. Aliphatics

Aliphatics are organic compounds containing carbon and hydrogen joined together in straight chains, branched chains, or non-aromatic rings. Aliphatics can be cyclic, but only aromatic compounds contain an especially stable ring of atoms. A number of aliphatics mostly fatty acids were identified from the Malvaceace members including Palmitic acid, Pinellic acid, Linoleic acid, Oleic acid, Steric acid (Table 1). Pinellic acid in methanol extract prepared from *A. indicum* was shown to have cytotoxic effect on U87MG human glioblastoma cells [71]. Palmitic acid extracted from *H. sabdariffa* flower was suggested to possess antioxidant activity [118]. Although role of other aliphatics have not been directly deciphered but hints from other plants can suggest their potential role as bioactive chemicals in the four plant species also.

### 3.2. Alkaloids

Alkaloids are a class of phytochemicals that contain basic nitrogen atom, although some alkaloids contain oxygen, sulfur and chlorine. Alkaloids are also a widely spread class of phytochemicals present in most of the medicinal plants. Alkaloids 1-lycoperodine and 1-methoxycarbonyl-β-carboline were reported from *A. indicum* [69] while O-Methylisourea hydrogen sulfate and β-sitosterol benzoate were identified in *H. sabdariffa* and the later was shown to have antioxidant property. Many pharmaceutical properties of *S. acuta* and *S. rhombifolia* are attributed to alkaloids. Cryptolepine isolated from *S. acuta* showed anticancer activity in human gastric adenocarcinoma (AGS) cells [98]. It also showed antimalarial and antimicrobial activities.Cryptolepine was also shown to be vasorelaxant in rat mesenteric artery rings [113]. In a study by Jang et al., cytotoxic activity of quinodolinone, crytolepinone and 11-Methoxyquindoline from *S. acuta* was shown using a mouse mammary organ culture model [101].

### 3.3. Phenolics

Plant phenolics are diverse in structure but are characterized by presence of hydroxylated aromatic ring. These are the largest category of phytochemicals and are widely distributed across the plant kingdom.Phenolic compounds, present in plant foods may be partly responsible for the pharmaceutical properties. Phenolic acids, polyphenols and flavonoids are the important groups of phenolics. Phenolic acids form a diverse group that includes the widely distributed hydroxybenzoic, hydroxycinnamic acids, chlorogenic acid and vanillic acid. More than 15 phenolics have been identified in *A. indicum* [119] (Table 1) and out of these, eugenol was shown to possess analgesic activity [68] while syringic acid and methyl caffeate were reported to be cytotoxic [74,75]. Ferulic acid in *H. sabdariffa* was shown to have antioxidant activity and hypoglycaemic effect in STZ-induced diabeticmice [90]. Chlorogenic acid is another phenolic acid present in both leaf and calyx extracts of *H. sabdarrifa* having anti-inflammatory, anti-mutagenic and anxiolytic properties [89]. Phenolics such as, evofolin A, evofolin B, *N*-trans-ferulolyltyramine, ferulic acid, sinapic acid, syringic acid and canillic acid were identified in the extract of the whole plants of *S. acuta* [101].

### 3.4. Flavonoids

Flavonoids are a class of plant phenolics which are further classified into several subclasses including anthocyanins, flavonols, flavanols, flavanones, flavones, and isoflavones [120]. Flavonoids are the largest group of plant phenols and the most studied.Abutilin A and quercetin were identified from *A. indicum* [69,72].A number of flavonoids were identified from *H.sabdariffa* with anthocyanins being a major subclass. Cyanidin-3-sambubioside and cyanidin-3-glucoside as the major compounds were reported in this plant [121]. Anthocyanins, cyanidin-3-glucoside, cyanidin-3-sambubioside, cyanidin-3-rutinoside, delphinidin-3-glucoside, delphinidin-3-sambubioside and delphinidin-3-xyloglucoside are the chief constituents of *H. sabdariffa* flowers and have shown antioxidant properties [75]. Other flavonoids identified from *H. sabdariffa* are protocatechuic acid, quercetin, hibiscetine, sabdaretine, gossypetine, hibiscetine, hibiscitrin and naringenin. Protocatechuic acid is an important phytochemical having antibacterial, hepatoprotective and anti-cancerous properties. Chiu et al. have reported anti-aging and anti-cancerous effects of naringenin [122]. Flavonoids kaempferol-3-o-α-l-rhamnopyranosyl-β-d-glucopyranoside and kaempferol-3-*O*-β-d-glucopyranoside were reported in *S. acuta* [98]. Flavonoid 5,7-dihydroxy-4′-methoxyflavone was identified for the first time in *S. rhombifolia* [113]. Recently, kaempferol and kaempferol-3-*O*-β-d-glycosyl-600-α-d-rhamnose were also identified in *S. rhombifolia* [114].

### 3.5. Steroids

All steroids have a characteristic chemical structure based around carbon atoms linked by single or double bonds and arranged into four interconnected rings. Steroidal compounds are pharmacologically important as many of them form sex hormones [123]. Stigmasterol and β-sitosterol are present in all the four plants under study. In *S. rhombifolia*, these were reported to have antibacterial properties [116,117]. Other important steroids present in these Malvaceae members include cholesterol, campesterol, clerosterol, Δ-5-avenasterol and spinasterol.

### 3.6. Peptides

Plant proteins and peptides with bioactivity are also a class of phytochemicals. Such peptides are often not hydrolysed in the digestive tract and have specific action in the body. Peptide, aurantiamide acetate and roseltidar T1 were identified as phytochemicals in *A. indicum* and *H. sabdariffa* respectively. Recently, Kam et al. [87] have reported that roseltide rT1, a bioenergetic-mitochondria-targeting peptide from *H. sabdariffa*, improves bioenergy traits by increasing cellular ATP level and therefore can be used for treating mitochondrial dysfunctions.

### 3.7. Ecdysteroids

Ecdysteroids are polyhydroxylated ketosteroids that are structurally similar to androgens. Phytoecdysteroids are plant-derived ecdysteroids, that plants synthesize for defense against pathogens (insects). These compounds are mimics of hormones used by arthropods in the molting [107]. Phytoecdysteroids ecdysone, 2D-hydroxyecdysone, 25-Acetoxy-20-hydroxyecdysone-*O*-β-d-Glucopyranoside, pterosterone-3-*O*-β-d-Glucopyranoside and ecdysone-3-*O*-d-β-d-Glucopyranoside were exclusively reported from *Sida* species only.

### 3.8. Terpenes

Terpenes are hydrocarbons of plant origin with general formula (C_5_H_8_)_n_ along with their oxygenated, hydrogenated and dehydrogenated derivatives. Terpenes are derived from isoprene chains and are classified according to the number of isoprene units. Vomifoliol, ioliolide, taraxast-1.20(30)-dien-3-one, taraxasterone and α-amyrine were reported to present in *S. acuta*. Of these vomifoliol, loliolide were shown to induce quinone reductase and to inhibit 7,12-dimethylbenz-[a]anthracene-induced preneoplastic lesions in a mouse mammary organ culture model [101].

### 3.9. Coumarins

Coumarins belong tobenzopyrene family consisting of a benzene ring joined to pyronering. A large number of coumarinsare present in plants. Umbelliferone, esculetin and scopoletin show a widespread presence in plant kingdom [124]. Scopoletin was reported from *A. indicum* [69], *S. acuta* [100] and *S. rhombifolia* [114]. Scoparone, p–coumaric, methylcoumarate and trans-p-coumaric acid were reported in *A. indicum* [69]. Heraclenol and escoporonewere reported from *S. acuta* [106] and *S. rhombifolia* [114] respectively.

### 3.10. Tocopherols

Tocopherols play an important role as antioxidants and also in maintaining membrane stability in plants [125]. α-Tocopherol is the major vitamin E compound found in leaf chloroplasts.α-tocopherol, γ-tocopherol and δ-tocopherol are reported to be present in *H. sabdariffa* [94]. α-Tocopherol, 7-Methyoxymethyl-α-tocopherol, β-Tocopherol and Tocospiro form *S. acuta*, are reported to have antioxidant activity [109].

## 4. Ethnopharmacological Activities

A summary of ethnopharmacological activities (based on scientific investigations) reported among the Malvaceae members (*A. indicum*, *H. sabdariffa*, *S. acuta* and *S. rhombifolia*) is represented in Table 2. A total of 31 major ethnopharmacological activities are listed (Table 2), of which ten (analgesic, anti-inflammatory, antidiabetic and antiobesity, antioxidant, antimicrobial, anxiolytic, cardioprotective, cytotoxic, hepatoprotective and nephroprotective) activities have been demonstrated in all four Malvaceae members. Seven ethnopharmacological activities have been reported in any one plant species (*A. indicum*: anti-asthmatic, increased fertility and anti-estrogenic; *H. sabdariffa*: anti-hyperammonemic, anti-hypertensive and anti-mutagenic; *S. acuta*: abortifacient). The ethnopharmacological activities of extracts/isolates of various plant parts of *A.indicum*, *H. sabdariffa*, *S. acuta* and *S. rhombifolia* are detailed in Table 3. The choice of plant part used for making the extract (for assessing ethnopharmacological activity) is extremely important In addition, age of the plant can affect the quantity of various classes of compounds, especially alkaloids and phenolics, and therefore is an important criterion to be considered while making plant extracts

### 4.1. Analgesic and Anti-Inflammatory

All four plant species exhibit anti-inflammatory and analgesic activities (Table 3). Ethanolic (EtOH) and methanolic (MeOH)/aqueous extracts of whole plant (WP) of *A. indicum* and aerial parts (AP)/roots of *S. rhombifolia* suppress carrageenan induced oedema in rats and the effect is comparable to ibuprofene [151] and indomethacin [220,223] respectively. Ethanolic extract of calyx of *H. sabdariffa* has demonstrated anti-inflammatory activity and anti-nociceptive activities by xylene-induced ear oedema and acetic acid writhing test in rat models [175]. Aqueous acetone extract of *S. acuta* showed analgesic effect in wistar mice model [199]. Diabetes related inflammation has been shown to reduce in mice models, when treated with acetone extracts of AP of *S. acuta* [196].

### 4.2. Antidiabetic and Antiobesity

Butanol extract of WP of *A. indicum* reduces insulin resistance in rodents by peroxisome proliferator activated receptor-gamma (PPAR-γ) agonist activity and enhancing glucose utilization [148]. Also, the plant extract was found be beneficial for reducing insulin resistance owing to its potential of controlling adipocyte differentiation and elevating utilization of glucose by enhancing promoter activity of Glucose transporter 1 (GLUT1) [148]. Aqueous and alcohol extracts of leaf of *A. indicum* promoted insulin production in moderately diabetic rats [145]. The calyx extract (aqueous) of *H. sabdariffa* is shown to prevent streptozotocin-induced liver injury in diabetic rats [168]. In *S. acuta*, the leaf extract exhibited hypoglycaemic and hypolipidaemic effectson alloxan-induced diabetic rats [193]. Methanolic extract of *S. rhombifolia* decreased blood glucose and hence demonstrated anti-hyperglycaemic effect in streptozotocin-induced diabetic rats [228].

### 4.3. Antimicrobial

The leaf extracts of A.indicum in chloroform, water and ethanol have shown anti microbial activity against Escherichia coli, Bacillus subtilisin, Staphylococcus aureus, Klebsiellapneumoniae, Salmonella typhi, Pseudomonasaeruginosa, Aspergillus niger and Candida parapsilosis [130,131]. The calyx extract of *H. sabdariffa* showed antibacterial effect against several bacteria including oral cavity bacteria, Streptococcus aureus and Micrococcus lutens [166]. However, no antifungal effect was observed against Candida albicans. Ethanolic extract showed better antimicrobial activity compared to aqueous extract of calyx of *H. sabdariffa*. It has been suggested that EtOH extract and protocatecchuic acid can be useful in food industry for preventing microbial contaminants [233]. In S. acuta, flavonoid and alkaloid extracts have shown antimicrobial activity. Strong antifungal activity against C. albicans was observed in the flavonoid fraction of most parts of *S. acuta* [208]. Methanolic, EtOH, chloroform extracts and alkaloid fractions of various plant parts of *S. rhombifolia* exhibited antimicrobial activity (Table 3). All investigations on antimicrobial activity were in vitro and by disc diffusion method [215,229,231]. Stigmasterol and β- sitosterol from root extract of *S. rhombifolia* have been identified as the antimicrobial compounds in the plant [117].

### 4.4. Antioxidant

The antioxidant activity of *A. indicum* has been correlated with the total content of phenols and flavanols. Ethylacetate extract showed maximum free radical scavenging activity compared to chloroform, petroleum ether, butanol, aqueous and ethanol extracts [134]. In *H. sabdariffa*, extracts of leaf [156,159], calyx [168] and flower [20] showed antioxidant effect in mice models. The extracts have scavenging effect on reactive oxygen and free radicals [163,234]. Further, the antioxidant activity is associated with inhibition of xanthine oxidase (XO) activity, protection from oxidative damage [163], increased levels of superoxide dismutase, catalase and glutathione and decreased malondialdehyde in liver [235]. Acetone extract of WP of *S. acuta* exhibited antioxidant activity in DPPH (2,2-diphenyl-1-picrylhydrazyl) and XO inhibition assays [109,202]. In *S. rhombifolia*, antioxidant activity in MeOH extract of leaves [217] and EtOH extracts of roots, stem, leaf and WP in DPPH, superoxide, NO and lipid peroxidation assays have been reported [221].

### 4.5. Cardioprotective and Anti-Hyperlipidemic

Ethanolic extract of roots of *A. indicum* exhibited cardioprotective effect against isopropanol- induced myocardial infarction in male rats [141]. Oral administration of EtOH extract for 28 days in rats, significantly prevented cardiovascular dysfunction. Also, a significant fall was reported in levels of serum marker enzymes (including creatine kinase-MB, Aspartate, Transaminase, Alanine transaminase and lactate dehydrogenase) in rats administered with root extract compared to isopranol-administered rats. In the same study, increased antioxidant parameters were reported in heart homogenate, indicating that cardioprotective effect might be related to the antioxidant activity of the plant extract. The aqueous and EtOH extracts of leaf of *A. indicum* caused lowering of elevated cholesterol and triglyceride in Triton WR1339 administered rats [146]. In another study, hydro-ethanolic extract of the plant reduced the level of triglycerides, TC, LDL and VLDLup to 20.64% and 43.8%, 39.83% and 20.63% respectively [150]. Administration of aqueous extract of petals of *H. sabdariffa* to hypertensive rats helped in reversing cardiac hypertrophy [182]. Extracts of calyx and leaf also reduced lipids, thus preventing cardiovascular diseases [156]. Methanolic extract of WP of *S. acuta* reduce heart beat rate and blood flow in cardiac vessels [204]. Ethanolic extract of leaf of *S. rhombifolia* has significant cardioprotective effect on isoproterenol induced myocardial necrosis in rats [212].

### 4.6. Hepatoprotective

Paracetamol and carbon tetrachloride-induced hepatotoxicity in rats could be reversed by aqueous extract of *A. indicum* [129]. Anthocyanin-rich, EtOH extract of calyx of *H. sabdariffa* exhibited hepatoprotective effect on thioacetamide induced hepatotoxicity in rats [180]. Root extract of *S. acuta* significantly decreased bilirubin, SGPT and SGOT values in paracetamol-induced hepatotoxicity in rats [194]. Powdered root, MeOH and aqueous extract of AP of *S. rhombifolia* has hepatoprotective effect against CCl_4_-induced hepatotoxicity in rats [223].

### 4.7. Nephroprotective

Ethanolic extract of roots of *A. indicum* showed nephroprotective effect in gentamicin-induced acute renal failure in rats [140]. Aqueous extract of calyx of *H. sabdariffa* significantly decreases the effect of adenine-induced chronic kidney disease (CKD) in rats. Infusion in the form of tea is taken in many parts of the world and therefore its nephroprotective effect adds to its dietary value [171]. In vitro experiments with MeOH and aqueous extracts of roots of *S. acuta* have shown to inhibit kidney stone (calcium oxalate crystals) growth [195]. In *S. rhombifolia*, in vivo experiments have shown that leaf extract has nephroprotective effect in gentamicininduced nephrotoxicity in rats by decreasing urea and creatine in urine along with an increase of renal antioxidants [213].

### 4.8. Anxiolytic 

Anxiety and hypertension are often treated together. Alcoholic leaf extract of *A. indicum* was tested for anti-anxiety property on rats at a dose of 400 mg/kg [127]. The Elevated Pulse Maze (EPM) was used for measuring the anxiety in control and experimental albino mice. The mice treated with an oral dose of alcoholic leaf extract showed less anxiety compared with the control group. Ethanolic extract of dried calyces of *H. sabdariffa* exhibited anxiolytic effect in animal models using EPM test. Increased anxiolytic and sedative effect was also observed with repeated administration of the extract doses [179]. In human beings, during clinical trials, aqueous extract of anthocyanin from calyx of *H. sabdariffa* was administered to patients with hypertension. The anti-hypertensive effect was then compared with control group who were given catopril. The results showed no difference in anti-hypertensive activity between experimental and control group indicating effectiveness of calyx extract to reverse hypertension [172]. Adminstration of leaf and stem extracts of *S. acuta* and EtOH extract of WP of *S. rhombifolia* to mice in EPM experiment showed anxiolytic effect on mice [189,230]. The extract also had sedative effect on mice which led to anti-anxiety (relaxed) state in the experimental mice [189].

### 4.9. Cytotoxicity

Petroleum ether, methanol, chloroform and ethyl acetate fractions of *A. indicum* exhibited cytotoxic activity on U87MG human glioblastoma cells. Maximum activity was observed in a sub fraction of chloroform extract, which yielded four different components inpurified form through repeated chromatography. These components were methyl caffeate, syringic acid, trans-p-courmarate and pinellic acid. Methyl caffeate was found to be relatively more active [71]. The hydromethanolic leaf extract of *A. indicum* has been shown to reduce the growth and viability of *Schizosaccharomyces pombe* cells and the active compound responsible for cytotoxicity was identified to be phytol [152]. Aqueous methanolic extract of calyces of *H. sabdariffa* showed cytotoxic effect in brine shrimo lethality assay [166]. In *S. acuta* and *S. rhombifolia*, in vitro studies demonstrated cytotoxic activity in extracts of WP, AP, and/or leaf [101,203]. Three alkaloids (quindolinone, cryptolepinone and 11-methoxy quindoline) isolated from *S. acuta* showed significant cytotoxicity in mouse hepatoma cells [101]. Another alkaloid, cryptolepine isolated from *S. acuta* showed strong cytotoxicity to TRAIL (Tumor necrosis factor Related Apoptosis-Inducing Ligand)-sensitive human gastric adenocarcinoma cells [98].

### 4.10. Anticancer and Anti-Proliferative

The ethanolic leaf extract of *A. indicum* showed anti-proliferative activity on cancer cell line by inducing the gene of apoptosis- activating factor (Apaf-1) through a network of various proteins [236]. Gold nanoparticles of leaf extract of *A. indicum* induces apoptosis in colon cancer cells [136]. Aqueous extracts of leaf and calyx of *H. sabdariffa* have anticancerous effects. Calyx extract has shown chemopreventive effect on human gastric carcinoma [162]. Aqueous extract of WP of *H. sabdariffa* has cytostatic effect on multiple myeloma cells and oral squamous cell carcinoma, thus indicating antitumor activity [184]. Cryptolepine isolated from *S. acuta* showed strong activity in overcoming TRAIL-resistance in human gastric adenocarcinoma (AGS) cells [98].

### 4.11. Anti-Diarrheal

Aqueous and MeOH extract of leaf of *A. indicum* exhibited significant anti-diarrheal activity in castor oil-induced and prostaglandin E2-induced diarrhoea in rats compared to the standard drug loperamide [133]. Calyx extract of *H. sabdariffa* also exhibits anti-diarrheal activity in castor oil-induced diarrhoea in rats [175]. Methanolic extract of roots of *S. rhombifolia* could treat diarrhoea in castor oil-induced diarrhoea in mice models [222].

### 4.12. Immuno-Stimulatory Activity

When aqueous and EtOH extracts of leaf of *A. indicum* were orally administered to experimental mice, an immune-stimulating effect was observed in the animals [237]. Aqueous EtOH extract of calyx of *H. sabdariffa* showed higher immune-stimulatory effect in comparison to the drug levamisole. The extract stimulated production of interleukin-10 and lowered the production of TNF-α in mouse model [179]. The extract may be tried as immune-stimulatory agent in humans.

### 4.13. Anticonvulsant and Neuroprotective

The aqueous and EtOH leaf extracts of *A. indicum* exhibited anticonvulsant activity against pentylene tetrazole (PTZ) and Maximal Electro Shock (MES) induced convulsion in Wistar rats. An oral dose of extract (100 mg/kg and 400 mg/kg) could protect the rats against induced convulsions [132]. Further, chloroform extract has been shown to be more potent than aqueous and EtOH leaf extracts [238]. In case *of H. sabdariffa*, aqueous extract of calyx prevent lipid-peroxidation in pro-oxidant induced lipid peroxidation in rat brain cells, thus suggesting a neuroprotective role. This activity could be attributed to high phenolic content resulting in strong antioxidant properties of the extract [173]. In *S. acuta*, leaf and stem extracts have anticonvulsant effects on pentylene tetrazole (PTZ)- induced seizures in mice [189]. Ethanolic extract of leaf of *S. acuta* showed neuroprotective effect in cerebral cells of experimental rats [239].

### 4.14. Antiulcer

Leaf extract of *A. indicum* showed significant antiulcer activity in asprin plus pyrolus-induced, ethanol-induced and acetic acid-induced ulcers in rat models. The treatment with extract indicated higher gastroprotective activity when compared to famotidine [128]. Oral administration of EtOH extract of calyx of *H. sabdariffa* in indomethacin-induced gastric ulcer showed antiulcer effect in Wistar Albino rat models [178]. In vivo experiments with extracts of *S. acuta* in rats models, indicated significant antiulcer activity compared to reference ulcer drug famotidine [36].

### 4.15. Antivenom

Methanolic leaf extract of *A. indicum* could inhibit the activity of enzymes present in the venom of *Echis carinatus* (Indian saw scaled viper) [139]. In vitro experiments with extract demonstrated suppression of activity of protease, phospho-monoesterase, phosphodiesterase, acetylcholinesterase, phospholipase A2, hyaluronidase and Lamino acid oxidase of snake venom. Ethanolic extract of WP of *S. acuta* can neutralize the venom of *Bothraps atrax* [201].

### 4.16. Anti-Arthritic

The plant extract of *A. indicum* has been tested in vitro for anti-arthritic activity which showed a dose dependant effect on protein denaturation, membrane stabilization and inhibition of proteinases. The herbal extract exhibited more potent analgesic activity than acetyl salicylic acid, a well-established analgesic drug, for arthritis [144]. Ethanolic extracts of root and stem of *S. rhombifolia* exhibited significant anti-arthritic effect in vivo, using adjuvant-induced arthritis in rat model [219].

### 4.17. Antipyretic

Ethanolic extract of calyx of *H. sabdariffa* and leaf of *S. acuta* have antipyretic effect on yeast-induced fever in rats [177,188]. It was suggested that calyx extract of *H. saddariffa* inhibits the formation of interleukin, interferons and tumor necrosis factor-α, which are produced during fever.

### 4.18. Anti-Atherosclerotic

Anthocyanin rich extracts from *H. sabdariffa* inhibit low density lipoprotein (LDL) oxidation and slow down the progression of atherosclerosis by preventing lipid accumulation in rabbits fed with high cholesterol diet [165].

### 4.19. Antispasmodic/Anticholinergic

Aqueous extract of calyx of *H. sabdariffa* shows antispasmodic effect in muscle preparations, e.g., rabbit aortic strip, rat uterus and rat diaphragm [174]. In *S. rhombifolia*, n-hexane extract of whole plant showed has strong anti-cholinesterase activity, thus suggesting its anticholinergic role [232].

### 4.20. Antigout

In *S. acuta*, dichloromethane and ethylacetate fractions of WP extract showed antigout activity in XO inhibitory assay [202]. In *S. rhombifolia*, the flavonoid fraction from extract of AP showed significant antigout effect by XO activity inhibition.

### 4.21. Antiplasmodial

Aerial parts of *S. acuta* contain cryptolepine which shows antiplasmodic effect against *Plasmodium falciparum* [96]. Methanolic extract of leaf of *S. rhombifolia* showed antiplasmodial activity against *Plasmodium bergheii* infection in mice [218].

### 4.22. Anti-Asthmatic

Methanolic extract of aerial parts of *A. indicum* showed mast cell stabilization in egg albumin- induced mast cell degranulation, in rat peritoneum. In the same study, anti-inflammatory effect was observed in carageenan-induced rat paw oedema model. It was suggested that bronchial asthma could be treated because of mast cell stabilization and anti-inflammatory effects of the plant extract [142].

### 4.23. Abortifacient 

Ethanolic leaf extract of *S. acuta* showed significant anti-implantation activity in pregnant rats up to 7 days after conception [190].

### 4.24. Anti-Estrogenic Activity

Methanolic plant extracts of *A. indicum* were tested for uterotropic and uterine peroxidise activities in ovariectomized rats and a negative correlation was established between these parameters and the plant extract. The plant extract could significantly reduce the activity of these enzymes and uterotropic response in estradiol treated rats [143].

### 4.25. Anti-Hyperammonemic

Oral administration of alcoholic extract of *H. sabdariffa* to ammonium chloride-induced, hyperammonemic rats, reduces ammonia, urea, uric acid, creatinine and non-protein nitrogen to normal levels in blood, indicating its anti-hyperammonemic effect [157].

### 4.26. Anti-Mutagenic

Aqueous extract of calyx of *H. sabdariffa* showed chemoprotective effects in cyclophosphamide- induced DNA damage in male Wistar rats, thus indicating anti-mutagenic activity against chemical (cyclophosphamide)- induced carcinogenesis [169].

### 4.27. Antitubercular

Methanolic extract of WP of *H. sabdariffa* [185] showed in vitro antitubercular activity against the strains *clinical* and *H37Rv* of *Mycobacterium tuberculosis*. Root extract of *S. rhombifolia* showed effectiveness against the standard strain *M. tuberculosis* H37Rv [216]. The ethylacetate leaf extract of *S. rhombifolia* is effective against a strain of *M. tuberculosis* which was resistant to streptomycin, isoniazid, rifampicin and ethambutol, thus emphasizing its antitubercular activity [216].

### 4.28. Antiviral

The leaf ethanolic extract of *H. sabdariffa* showedin vitroantiviral effect against virus extract consisting of Hep-2 cells [161]. Methanolic extract of leaf of *S. acuta* showed antiviral activity against *Herpes simplex* virus in virus-induced cytopathic assay [192].

### 4.29. Anti-Hypertensive and Vasorelaxant

Aqueous extract of petals and the crude MeOH extract of calyces of *H. sabdariffa* could relax aortic rings of muscles of hypertension-induced rats, thus demonstrating a vasorelaxant effect [32,181]. The alkaloid fraction of *S.rhombifolia* showed vasorelaxant activity in rat mesenteric arterial rings. Cryptolepinone, the compound isolated from the alkaloid fraction is shown to have vasorelaxant activity [113]. The alkaloids quinodolinone and salt of crytolepine also have vasorelaxant effect [114].

## 5. Toxicity Studies

Toxicity testing is done to obtain information on the biological activity and mechanism of action of the drug. The information generated by the test is used to assess safety of the drug. Toxicity of any compound is measured in the terms of LD50 which is standard measure of toxicity of a substance that is sufficient to kill half of the sample population of a test animal. Acute toxicity of dried powder of aerial parts as well as fresh juice of leaves of *A. indicum* was measured in Swiss mice. Administration of either of the above plant material did not show any significant effect on body weight [240]. Acute oral toxicity of the aqueous extract and aqueous suspension of the ethanolic extract of *A. indicum* leaves was measured in Swiss albino mice. These were found to be safe at dose of 4000 mg/kg and 2000 mg/kg respectively and did not show mortality in mice [237]. In mice, toxicity was not observed within 7 days after oral administration at the dose of 15 g/kg of ethanol and aqueous extracts of *H. sabdariffa* calyces [177]. Effects of oral administration of water and alcohol extracts of dried calyx of *H. sabdariffa* for 90-day were examined in albino rats. A dose of 2000 mg/kg caused the death of the animals which was preceded by a severe weight loss [241]. Sireeratawong et al. [242] measured the toxicity study of water extract from the calyces of *H. sabdariffa* by single and long-term oral administration in rats. The results indicated that the single oral administration of extract in the amount of 5000 mg/kg body weight does not produce acute toxicity.

For determining acute toxicity test of *S. acuta*, mice were injected with aqueous acetone extract of dried plant material at dose of 1; 2; 2.5; 3; 4; 5 and 6 g/kg and the LD50 values of 3.2 g/kg was determined, suggesting that the extract has negligible level of toxicity when administered orally [199].While ethanolic extract of *S. acuta* was suggested to be toxic when administered at a dose of 200 mg/kg to Wistar rats [243]. Acute toxicity of the aqueous-methanol extract of *S. rhombifolia* was measured using Albino wistar rats. The animals exhibited slight changes in general behaviour but did not expressed changes in their physio-pathological activities [231].

## 6. Conclusions and Future Prospects

This review presents scientific investigations thatjustify (i) the use of plant extracts of 4 Malvaceae members (*A. indicum*, *H. sabdariffa*, *S. acuta* and *S. rhombifolia*) by Indian tribal populations; and (ii) their use as ingredients in Indian traditional medicines. The pharmacological activities of the extracts and isolates of these plants that have been investigated, can be correlated with the traditional ethnomedicinal uses, detailed in Table 4, and in some cases the active key compound has also been identified. All four ethnomedicinal plants have some common classes of phytoconstituents (alkaloids, phenolics, flavonoids and steroids) to which many of the ethnopharmacological activities can be attributed. The age of the plant and the plant part used for extraction are important parameters, which can affect the ethnopharmacological activity of the extract. In case of alkaloids, older plants have much less alkaloids compared to the younger plants. Likewise, aerial parts of *S. acuta* contain good quantity of crytolepine and quindoline whereas these two compounds are absent from aerial parts of *S. rhombifolia.* It is also observed that many ethnopharmacological activities (anti-inflammatory, analgesic, cytotoxic, etc.) are common to all four plants (see Table 2). Since all four plants belong to same taxonomical family (Malvaceae) and also show several common ethnopharmacological activities, identification of the active principle in one plant (e.g., eugenol identified in *A. indicum* has an analgesic effect) can help in assessing the presence of that compound in the rest of the plants. Some of the phytoconstituents are common between two or more of the four Malvaceae members, but their reported ethnopharmacological effects are different (e.g., ferulic acid from *H. sabdariffa* has anti-diabetic and anti-ageing effects whereas, ferulic acid from *S. acuta* has hepatoprotective effect). This suggests that the phytochemicals in separate sets of conditions can exhibit different pharmacological activities which may be due to complex interaction of the phytochemicals in the cells/body of the organisms. Therefore, further studies may be undertaken to understand the exact mechanism of action of different phytoconstituents showing various pharmacological activities, by taking cue from existing scientific investigations.

## Figures and Tables

**Table 1:** Major phytoconstituents identified from *Abutilon indicum*, *Hibiscus sabdariffa*, *Sida acuta* and *Sida rhombifolia*.

Plant	Phytoconstituents		Pharmacological Properties	References
	Class	Type		
***Abutilon indicum***	**Acid**	Fumaric acid(organic acid)		[68]
		Galacturonic acid (sugar acid)		[68]
		Methyl indole-3-carboxylate (acid)		[69]
	**Alcohol**	Hinesol		[70]
		Cubenol		[70]
		Phytol		[70]
		Gamma-sitosterol Lupeol		[70]
	**Aliphatics**	Palmitic acid		[70]
		Pinellic acid	Cytotoxic	[71]
	**Alkaloids**	1-lycoperodine		[69]
		1-methoxycarbonyl-β-carboline		[69]
	**Alkane Hydrocarbon**	Tertacontane		[70]
		n-tetracosane		[70]
		All-trans-squalene		[70]
	**Aromatic ketone**	3-hydroxy-β-damascone		[69]
		3-hydroxy-β-ionol		[69]
	**Coumarins**	Scopoletin		[69]
		Scoparone		[69]
		p–coumaric		[68]
		Methylcoumarate		[69]
		Trans-p-coumaric acid		[69]
	**Flavonoid**	Abutilin A		[69]
		Quercetin		[72]
	**Peptide**	Aurantiamide acetate		[69]
	**Phenolic**	Eugenol [4-allyl-2-methoxyphenol]	Analgesic activity	[73]
		Syringic acid	Cytotoxic	[71]
		Benzoic acid		[69]
		Vanillic acid		[68,69]
		Gallic acid		[72]
		N-feruloyl tyrosine		[69]
		Caffeic acid		[69]
		p-β-d-glucosyloxybenzoic acid		[68]
		4-hydroxy-3-methoxy-trans-cinnamic acid methyl ester		[69]
		Methyl caffeate	Cytotoxic	[71]
		p-hydroxybenzaldehyde		[69]
		Vanillin		[69]
		Syringaldehyde		[69]
		4-hydroxyacetophenone		[69]
		Methylparaben		[69]
	**Steroids**	β-sitosterol,		[69]
		Stigmasterol		[69]
		(R)-*N*-(1′-methoxycarbonyl-2′-phenylethyl)-4-hydroxybenzamide		[69]
	**Tocopherol**	di-alpha-tocopherol		[70]
***Hibiscus sabdariffa***	**Acid**	Malic acid		[74]
		Tartaric acid		[74]
	**Aliphatics**	Linoleic acid		[75]
	**Alkaloids**	β-sitosterol benzoate		[76]
	**Flavonoids**	Protocatechuic acid	Antibacterial	[77]
			Hepatoprotective	[78]
			Anti-cancerous	[79]
		Quercetin	Neuroprotective	[80]
		Hibiscetine		[81]
		Sabdaretine		[82]
		Gossypetine		[82]
		Hibiscitrin		[81]
		Naringenin	Anti-aging,	[83]
			Anti-cancerous	[83]
		Rutin		[84]
		Isoquercitin		[84]
		Kaempferol-3-o-rutinoside		[84]
		Kaempferol-3-o-glucoside		[84]
		Kaempferol		[84]
		Myricetin 3-arabinogalactoside		[85]
		Cyanidin-3-glucoside	Antioxidant	[86]
		Cyanidin-3-sambubioside	Antioxidant	[86]
		Cyanidin-3-rutinoside	Antioxidant	[86]
		Delphinidin-3-glucoside	Antioxidant	[86]
		Delphinidin-3-sambubioside	Antioxidant	[86]
		Delphinidin-3-xyloglucoside	Antioxidant	[86]
	**Peptide**	Roseltidar T1 (plant knottins)	Prevents mitochondrial dysfunction	[87]
	**Phenolics**	Neochlorogenic acid		[88]
		Chlorogenic acid	Antioxidant	[89]
			Antihyperlipidemic	[89]
		Cryptochlorogenic acid		[88]
		Ferulic acid	Anti-ageing,	[90]
			Antidiabetic	[90]
		Coumaroylquinic acid		[88]
		Hibiscus acid		[88]
		Caffeoylshikimic acid		[91]
		Eugenol		[92]
	**Polysaccharides**	HSP-II (Glucuronic acid, Rhamnose, Mannose, Glucose, Galactose)	Immunomodulation: Immune-enhancement	[93]
		Mannose, Sucrose, Xylose,		[75]
		α-Terpinyl acetate		[75]
	**Steroids**	Cholesterol		[94]
		Campesterol		[94]
		β-sitosterol		[94]
		Clerosterol		[94]
		Δ-5-avenasterol		[94]
	**Tocopherols**	α-tocopherol		[94]
		γ-tocopherol		[94]
		δ-tocopherol		[94]
***Sida acuta***	**Alkaloids**	Ephedrine		[95]
		Cryptolepine	Antimalarial	[96]
			Antimicrobial	[95,97]
			Cytotoxic	[98,99,100]
		Quinodolinone	Cytotoxic	[101]
		Crytolepinone	Cytotoxic	[101]
		11-Methoxyquindoline	Cytotoxic	[101]
		Quindoline	Antimicrobial	[95,99]
		Vasicinone		[102]
		Vasicine		[102]
	**Aliphatics**	Hentriacontane		[103]
		Nonacosane		[103]
		Pristane		[103]
		Phytane		[103]
		Sterculic acid		[104]
		Malvalic acid		[104]
		Myristic acid		[105]
		Palmitic acid		[105]
		Stearic acid		[105]
		Oleic acid		[105]
		Linoleic acid		[105]
	**Coumarins**	Scopoletin		[100,101]
		Heraclenol		[106]
	**Ecdysteroids**	2D-Hydroxyecdysone		[107]
	**Flavonoids**	Kaempferol-3-*O*-α-l-rhamnopyranosyl-β-d-glucopyranoside		[98]
		Kaempferol-3-*O*-β-d-glucopyranoside		[98]
	**Lignans**	4-Ketopinoresinol		[101]
		Syrigaresinol		[101]
		Acanthoside B		[106]
	**Phenolics**	*N*-trans-Ferulolyltyramine	Cytotoxic	[101]
		Evofolin A	Cytotoxic	[101]
		Evofolin B	Cytotoxic	[101]
		Ferulic acid	Hepatoprotective	[101]
		Sinapic acid		[101]
		Syringic acid		[101]
		Vanillic acid		[101]
	**Pthalate**	Di-(2-ethylhexyl)phthalate		[108]
	**Steroids**	Cholesterol		[103]
		Campesterol		[103]
		β-Sitosterol		[103]
		Stigmasterol		[103]
		Stigmast-7-enol(=22-dihydrospinasterol)		[103]
	**Terpenoids**	Vomifoliol	Cytotoxic	[101]
		Loliolide	Cytotoxic	[101]
		Taraxast-1.20(30)-dien-3-one		[109]
		Taraxasterone		[109]
		α-amyrine		[97]
	**Tocopherols**	α-Tocopherol	Antioxidant	[109]
		7-Methyoxymethyl-α-tocopherol	Antioxidant	[109]
		β-Tocopherol	Antioxidant	[109]
		Tocospiro	Antioxidant	[109]
***Sida rhombifolia***	**Aliphatics**	n-Hexacos-11-enoic acid	Antimicrobial	[110]
		Sterculic acid		[104]
		Malvalic acid		[104]
		Myrstic acid		[111]
		Palmitic acid		[111]
		Stearic acid		[111]
		Oleic acid		[111]
		Linoleic acid		[111]
	**Alkaloids**	β-Phenethylamine		[112]
		Ephedrine		[112]
		γ-(Pseudo)-Ephedrine		[112]
		N-methyl-β-Phenethylamine		[112]
		S-(+)-N_2-_Methyltryptophan methyl ester		[112]
		Hypophorine methyl ester		[112]
		Vascicine		[112]
		Crytolepinone	Vasorelaxant	[113]
		Salt of Cryptolepene	Vasorelaxant	[114]
	**Coumarins**	Scopoletin		[114]
		Escoporone		[114]
	**Ecdysteroids**	Ecdysone		[115]
		2D-Hydroxyecdysone		[115]
		2-Deoxy-2D-hydroxyecdysone-3-*O*-β-d-Glucopyranoside		[115]
		2D-Hydroxyecdysone3-*O*-β-d-Glucopyranoside		[115]
		25-Acetoxy-20-hydroxyecdysone- *O*-β-d-Glucopyranoside		[115]
		Pterosterone-3-*O*-β-d-Glucopyranoside		[115]
		Ecdysone-3-*O*-d-β-d-Glucopyranoside		[115]
	**Flavonoids**	5,7-Dihydroxy-4-methoxy flavones (=Acacetin)		[113]
		Kaempferol		[114]
		Kaempferol-3-*O*-β-d-glycosyl-600 -α-d-rhamnose		[114]
	**Phaeophytins**	Phaeophytin A		[113]
		13^2^-Hydroxy Phaeophytin B		[113]
		17^3^-Ethoxy phaeophorbide B		[113]
	**Phenolics**	Ethoxy-ferulate		[114]
	**Steroids**	Cholesterol		[116]
		Campesterol		[116]
		β-Sitosterol	Antibacterial	[116,117]
		Stigmasterol	Antibacterial	[116]
		Stigmast-7-enol(=22-dihydrospinasterol)		[116]
		22-Dehydrocampesterol		[116]
		Spinasterol		[116]
		24-Methylene cholesterol		[116]
		Sitosterol-3-*O*-β-d-Glucopyranoside		[113]
		Stigmasterol-3-*O*-β-d-Glucopyranoside		[113]

**Table 2:** Comparison of ethnopharmacological activities of *Abutilon indicum*, *Hibiscus sabdariffa*, *Sida acuta* and *Sida rhombifolia* (Y—Reported; N—Not reported).

S. No.	Ethnopharmacological Activity	*A. indicum*	*H. sabdariffa*	*S. acuta*	*S. rhombifolia*
1	Antidiabetic/Hypoglycaemic &Antiobesity	Y	Y	Y	Y
2	Anti-inflammatory	Y	Y	Y	Y
3	Hepatoprotective	Y	Y	Y	Y
4	Analgesic	Y	Y	Y	Y
5	Antioxidant	Y	Y	Y	Y
6	Antimicrobial/Antibacterial	Y	Y	Y	Y
7	Nephroprotective	Y	Y	Y	Y
8	Cytotoxic	Y	Y	Y	Y
9	Cardioprotective/Anti-hyperlipidemic	Y	Y	Y	Y
10	Anxiolytic	Y	Y	Y	Y
11	Anti convulsant/Neuroprotective	Y	Y	Y	N
12	Antiulcer	Y	Y	Y	N
13	Antitubercular	Y	Y	N	Y
14	Anti-diarrhoea	Y	Y	N	Y
15	Anticancerous/Anti-proliferative	Y	Y	Y	N
16	Anti-arthritic	Y	N	N	Y
17	Antipyretic	N	Y	Y	N
18	Anti-spasmodic/anticholinergic	N	Y	N	Y
19	Antiplasmodial	N	N	Y	Y
20	Antiviral	N	Y	Y	N
21	Anti-hypertensive & Vasorelaxant	N	Y	N	Y
22	Antigout	N	N	Y	Y
23	Antivenom	Y	N	Y	N
24	Antiasthmatic	Y	N	N	N
25	Anti-atherosclerotic	N	Y	N	N
26	Increases Fertility	Y	N	N	N
27	Anti estrogenic activity	Y	N	N	N
28	Anti-mutagenic	N	Y	N	N
29	Anti-hyperammonemic	N	Y	N	N
30	Immunostimulatory	N	Y	N	N
31	Abortifacient	N	N	Y	N

**Table 3:** Ethnopharmacological activities of the extracts from different parts of *Abutilon indicum*, *Hibiscus sabdariffa*, *Sida acuta* and *Sida rhombifolia*.

Name of Plant	Plant Part Used	Extract	Ethnopharmacological Activity	Reference
***Abutilon indicum***	**Leaf**	**Alcohol**	Hypoglycemic	[126]
			Anxiolytic	[127]
		**Alcoholic and aqueous**	Antiulcer	[128]
		**Aqueous**	Hepatoprotective	[129]
			Antimicrobial	[130,131]
			Anticonvulsant	[132]
			Anti-diarrheal	[133]
		**Butanol**	Antioxidant	[134]
		**Chloroform**	Antimicrobial	[130,131]
		**Ethanol**	Anti-inflammatory	[135]
			Anticonvulsant	[132]
			Antimicrobial	[130,131]
		**Gold nanoparticles of leaf extract**	Anticancer	[136]
		**Hexane**	Larvicidal against *Aedesaegypti*	[137]
		**Loperamide**	Anti diarrheal	[133]
		**Methanol**	Larvicidal	[138]
			Antivenom	[139]
			Anti diarrhoeal	[133]
	**Root**	**Ethanol**	Nephroprotective	[140]
			Cardioprotective	[141]
	**Aerial part**	**Methanol**	Anti-asthmatic activity	[142]
			Anti-estrogenic activity	[143]
			Anti-arthritic activity	[144]
	**Whole Plant**	**Alcoholic**	Hypoglycemic	[126]
		**Aqueous**	Antidiabetic	[145]
			Hypoglycemic	[126]
			Anti-hyperlipidemic	[146]
		**Biogenic silver nanoparticles**	Antioxidant	[147]
			Antibacterial	[147]
			Cytotoxic effects	[147]
		**Butanol**	Antidiabetic	[148]
		**Chloroform**	Enhanced Fertility	[149]
		**Ethanol**	Anti-hyperlipidemic	[148,150]
		**Methanol**	Analgesic	[151]
			Anti-inflammatory	[151]
		**Phytol**	Cytotoxic	[152]
***Hibiscus sabdariffa***	**Leaf**	**Aqueous**	Anticancerous	[153]
			Anti-proliferative	[154]
			Hypoglycaemic	[155]
			Anti-hyperlipidemic Antioxidant	[156]
			Anti-hyperammonemic	[157]
			Anti-atherosclerotic	[158]
			Hepatoregenerative	[159]
			Antioxidant	[159]
		**Aqueous-ethanolic**	Lipid lowering effect	[160]
		**Ethanolic**	Antiviral	[161]
	**Flower**	**Aqueous**	Anti-gastric carcinoma	[162]
			Antioxidant	[20]
		**Ethanolic**	Antioxidant	[163]
		**Methanolic**	Antiapoptotic	[164]
			Anti-atherosclerotic	[165]
	**Calyx**	**Aqueous-methanolic**	Antibacterial	[166]
			Cytotoxicity	[166]
		**Aqueous-ethanolic**	Immuno-stimulatory	[167]
		**Aqueous**	Antidiabetic	[168]
			Antioxidant	[168]
			Anti-mutagenic	[169]
			Hypocholesterolaemic	[170]
			Nephroprotective	[171]
			Anti-hypertensive	[172]
			Neuroprotective	[173]
			Antispasmodic	[174]
		**Ethanolic**	Anti-nociceptive	[175]
			Anti-diarrheal	[175]
			Anti-hypertensive	[176]
			Antipyretic	[177]
			Anti-inflammatory	[177]
			Antiulcer	[178]
			Anxiolytic	[179]
			Hepatoprotective	[180]
		**Methanolic**	Vasorelaxant	[181]
	**Petals**	**Aqueous**	Anti-hypertensive	[182]
			Cardioprotective	[182]
			Vasorelaxant	[32]
	**Seed**	**Aqueous**	Antimicrobial	[183]
	**Whole plant**	**Aqueous**	Antitumoral	[184]
		**Methanolic**	Antitubercular	[185]
***Sida acuta***	**Leaf**	**Aqueous**	Antimicrobial	[186]
		**Chloromethane**	Antimicrobial	[187]
		**Ethanol**	Antimicrobial	[186,187]
			Antiulcer	[36]
			Antipyretic	[188]
			Anticonvulsant	[189]
			Anxiolytic	[189]
			Abortifacient	[190]
		**n-Hexane fraction**	Hepatoprotective	[191]
		**Methanol**	Antimicrobial	[192]
			Antiviral	[192]
			Antidiabetic	[193]
			Antiobesity	[193]
	**Root**	**Methanol**	Hepatoprotective	[194]
			Kidney stone treatment	[195]
	**Aerial parts**	**Acetone**	Antioxidant	[196]
			Anti-inflammatory	[196]
			Anti-hyperglycemic	[196]
		**Ethanol**	Antimicrobial	[186]
		**Methanol**	Antiplasmodial	[96]
	**Whole plant**	**Alkaloid fraction**	Antiplasmodial	[197]
		**Aqueous**	Antimicrobial	[198]
		**Aqueous Me_2_CO**	Analgesic	[199]
			Anti-inflammatory	[199]
		**Ethanol**	Antimicrobial	[198]
			Antiplasmodial	[197]
			Anti ulcer	[200]
			Antivenom	[201]
		**Ethyl Acetate**	Analgesic	[202]
		**Methanol**	Cytotoxic	[203]
			Cardiovascular and cardioprotective	[204]
			Wound healing	[205]
		**Polyphenol**	Antimicrobial	[206]
			Antioxidant	[206]
	**Root, Leaf, Stem, Bud**	**Alkaloid**	Antimicrobial	[207]
		**Flavonoid**	Antifungal	[208]
***Sidarhombifolia***	**Leaf**	**Aqueous**	Hepatoprotective	[209]
			Antidiabetic	[210]
			Antiobesity	[210]
		**Ethanol**	Antimicrobial	[211]
			Cardioprotective	[212]
			Nephroprotective	[213]
			Anti-inflammatory	[214]
		**Ethylacetate**	Antimicrobial	[215]
			Cytotoxic	[215]
			Analgesic	[214]
			Antitubercular	[216]
			Anti-inflammatory	[214]
		**Chloromethane**	Cytotoxic	[215]
		**Methanol**	Antimicrobial	[211]
			Antioxidant	[217]
			Antiplasmodial	[218]
			Anti-inflammatory	[214]
	**Stem**	**Ethanol**	Anti-arthritic	[219]
	**Root**	**Aqueous**	Anti-inflammatory	[220]
		**Ethanol**	Anti-inflammatory	[220]
			Anti-arthritic	[219]
			Antioxidant	[221]
		**Ethylacetate**	Antitubercular	[216]
		**Methanol**	Antidiarrheal	[222]
		**Powdered form**	Hepatoprotective	[223]
	**Fruit**	**Methanol**	Antimicrobial	[224]
	**Aerial parts**	**Acetone**	Antioxidant	[196]
			Anti-inflammatory	[196]
		**Aqueous**	Hepatoprotective	[223]
			Anti-arthritic	[225]
		**Alkaloid fraction**	Antimicrobial	[226]
		**Cryptolepinone**	Vasorelaxant	[113]
		**Ethanol**	Cytotoxic	[227]
			Anti-arthritic, antigout	[225]
		**Methanol**	Hepatoprotective	[223]
			Analgesic	[227]
			Anti-inflammatory	[223]
			Antidiabetic	[228]
	**Whole plant**	**Aqueous**	Antimicrobial	[229]
		**Ethanol**	Antioxidant	[221]
			Anti-anxiety	[230]
		**Ethylacetate**	Antimicrobial	[229]
		**Methanol**	Antimicrobial	[231]
		**n-hexane**	Anti-inflammatory	[232]
			Anticholinergic	[232]
			Cytotoxic	[232]

**Table 4:** Justification of traditional medicinal uses of *Abutilon indicum*, *Hibiscus sabdariffa*, *Sida acuta* and *Sida rhombifolia* supportedby scientific investigations of their ethnopharmacological activities and phytoconstituents (‘-‘ indicates not reported).

Plant	Traditional Medicinal Use	Ethnopharmacological Activity	Phytoconstituent Identified
***A. indicum***	Inflammations [10]	Anti-inflammatory [135]	-
	Ulcer [10]	Antiulcer [128]	-
	Diarrhoea [10]	Anti-diarrhoea [133]	-
	Wounds [11]	Antimicrobial, Antibacterial [147]	-
	Diabetes [12]	Antidiabetic, Hypoglycaemic [145]	-
	Joint pains and Arthritis [13]	Analgesic, Anti-arthritic [151]	-
	Liver disorders/Jaundice [13]	Hepatoprotective [159]	-
	Bronchitis [13]	Anti-asthmatic [142]	-
	Pains [13]	Analgesic [151]	Eugenol [73]
	Enhanced male fertility [19]	Enhanced semen production [149]	-
***H. sabdariffa***	Skin inflammations [21]	Anti-inflammatory [177]	Roseltidar T1 (plant knottins) [87] Chlorogenic acid [89]
	Diarrhoea [26]	Anti-diarrheal [175]	-
	Wounds [28]	Antibacterial, Antimicrobial [166]	Protocatechuic acid [77] Chlorogenic acid [89]
	Pains [28]	Anti-nociceptive [175]	-
	Liver disorders [20]	Hepato-regenerative [159] Hepatoprotective [180]	Protocatechuic acid [78]
	Kidney disorders [27]	Nephroprotective [171]	Quercetin [80]
	Cardiac diseases [31]	Cardioprotective [182]	-
	Nervous disorders [20]	Neuroprotective [173]	-
	Controlling Blood Pressure [31]	Anti-hypertensive, Vasorelaxant [127]	-
	Pyrexia [20]	Antipyretic [177]	-
	Cancers [20]	Anticancerous, Antitumoral [153]	Protocatechuic acid [79], Naringenin [83]
***S. acuta***	Testicular swelling [35]	Anti-inflammatory [196]	-
	Ulcers [24]	Antiulcer [200]	-
	Wounds and infections [39]	Antimicrobial [198]	Crytolepine, Quinodoline [96] n-Hexacos-11-enoic acid [110]
	Headaches [24]	Analgesic [199]	-
	Liver disorders [24]	Hepatoprotective [194]	Ferulic Acid [101]
	Urinary disorders [25]	Kidney stone treatment [195]	-
	Neurological disorders e.g., paralysis [20]	Neuroprotective [189]	-
	Fever [43]	Antipyretic [188]	-
	Abortifacient [41]	Abortion [190]	-
	Dandruff [25]	Antifungal [208]	-
	Malaria [24]	Antiplasmodial [96]	-
	Snake bites [25]	Antivenom [201]	-
	Gonorrhoea (herpes) [24]	Antiviral [192]	-
***S. rhombifolia***	Inflammations [46]	Anti-inflammatory [214]	-
	Diarrhoea [45]	Anti-diarrhoea [222]	-
	Abscess & Wounds [44]	Antimicrobial [211]	n-Hexacose-11-enoic Acid [110], β-sitosterol, Stigmasterol [116]
	Arthritis [45]	Anti-arthritic [219]	-
	Headaches [45]	Analgesic [214]	-
	Rheumatic pain [46]	Analgesic [214]	-
	Liver disorders [46]	Hepatoprotective [209]	-
	Urinary disorders [46]	Nephroprotective [213]	-
	Heart disease [49]	Cardioprotective [212]	-
	Tuberculosis [46]	Antitubercular [216]	-
	Gout [45]	Antigout [225]	-
